# Haplotype-resolved chromosome-level genome assembly of *Ehretia macrophylla*

**DOI:** 10.1038/s41597-024-03431-9

**Published:** 2024-06-05

**Authors:** Shiping Cheng, Qikun Zhang, Xining Geng, Lihua Xie, Minghui Chen, Siqian Jiao, Shuaizheng Qi, Pengqiang Yao, Mailin Lu, Mengren Zhang, Wenshan Zhai, Quanzheng Yun, Shangguo Feng

**Affiliations:** 1 Henan Province Key Laboratory of Germplasm Innovation and Utilization of Eco-economic Woody Plant, Pingdingshan University, Pingdingshan, 467000 China; 2 Kaitai-bio Company, Hangzhou, 310000 China; 3 Henan Forestry Vocational College, Luoyang, 471000 China; 4 Henan Senzhuang Cukang Agriculture and Forestry Technology Co., Ltd, Luoyang, 471000 China; 5 Kaitai Mingjing Genetech Corporation, Beijing, 100070, China; 6 College of Life and Environmental Science, Hangzhou Normal University, Hangzhou, 310036 China; 7 Zhejiang Provincial Key Laboratory for Genetic Improvement and Quality Control of Medicinal Plants, Hangzhou Normal University, Hangzhou, 310036 China

**Keywords:** Genome, Evolutionary genetics

## Abstract

*Ehretia macrophylla* Wall, known as wild loquat, is an ecologically, economically, and medicinally significant tree species widely grown in China, Japan, Vietnam, and Nepal. In this study, we have successfully generated a haplotype-resolved chromosome-scale genome assembly of *E. macrophylla* by integrating PacBio HiFi long-reads, Illumina short-reads, and Hi-C data. The genome assembly consists of two haplotypes, with sizes of 1.82 Gb and 1.58 Gb respectively, and contig N50 lengths of 28.11 Mb and 21.57 Mb correspondingly. Additionally, 99.41% of the assembly was successfully anchored into 40 pseudo-chromosomes. We predicted 58,886 protein-coding genes, of which 99.60% were functionally annotated from databases. We furthermore detected 2.65 Gb repeat sequences, 659,290 rRNAs, 4,931 tRNAs and 4,688 other ncRNAs. The high-quality assembly of the genome offers a solid basis for furthering the fields of molecular breeding and functional genomics of *E. macrophylla*.

## Background & Summary

*Ehretia macrophylla* Wall is a perennial shrub tree belonging to the genus *Ehretia* in the Boraginaceae family. It can arrive at 15 m and is widely distributed in the southwest, south, and east of China, as well as in certain regions of Japan, Vietnam, and Nepal^1–3^. *E. macrophylla*, also known as wild loquat in China, is a rare tree with diverse applications, including ecological, gardening, ornamental, and medicinal value. To date, the complete sequencing of any species within the genus *Ehretia* remains unaccomplished. The genetic studies of *E. macrophylla* are impeded due to the absence of high-quality reference genome sequences, despite its multifarious applications.

*E. macrophylla* is an excellent tree species for urban greening and as a border tree, especially when dust retention is necessary. This is due to its high trunk, strong dust absorption ability, and resistance to pests and diseases^2^. Furthermore, the foliage of *E. macrophylla* serves a dual purpose as both a potential food source and medicinal resource, highlighting its multifaceted utility in various fields^4^. It has the effect of activating the meridians and treating rheumatism, dispelling wind and dampness, and relieving joint pain. Furthermore, the bark of *E. macrophylla* has the effect of dissipating blood stasis and swelling, making it suitable for treating fall injuries^3^. Of additional interest, the fruit of *E. macrophylla* serves as a functional food supplement, consumed as a traditional fruit and utilized in herbal tea. It can help soothe the throat and alleviate coughs. The fruit is usually used to treat diseases such as bronchitis, acute and chronic pharyngitis, cough, and asthma^2,5^. As a prominent species within the genus *Ehretia*, *E. macrophylla* is renowned for its diverse range of applications attributed to the copious presence of bioactive compounds in its fruit and other tissues. These bioactive substances remarkable antioxidant, antitumor, anti-inflammatory, antiviral, and antibacterial properties. Some of the key compounds found in *E. macrophylla* include quercetin, flavonoids, kaempferol, rosmarinate, caffeic acid, and pectin polysaccharide^2,4,5^.

High-quality genomes are of profound significance for in-depth research, rational development, and adequate protection of plants. Here, we present a high-quality genome assembly of *E. macrophylla* using an integrated approach, which includes PacBio HiFi long-read sequencing, short-read Illumina sequencing, and Hi-C sequencing. The assembled genome (~3.40 Gb) comprises haplotype a (1.82 Gb) and haplotype b (1.58 Gb), with contig N50 lengths of 28.11 Mb and 21.57 Mb, respectively. Furthermore, the assembled scaffolds were meticulously anchored to 40 pseudochromosomes with an exceptional anchoring rate of 99.41%. We predicted a total of 58,886 protein-coding genes, with 29,805 for haplotype a and 29,081 for haplotype b. Among these genes, 99.60% were functionally annotated. In addition, we identified 2.65 Gb repeat sequences (1.44 Gb for haplotype a and 1.21 Gb for haplotype b), and annotated a total of 668,909 non-coding RNA genes, including 659,290 rRNA (415,016 for haplotype a and 244,274 for haplotype b), 4,931 tRNA genes (2,522 for haplotype a and 2,409 for haplotype b) and 4,688 other ncRNA genes (2,428 for haplotype a and 2,260 for haplotype b). Our data will serve as a valuable genetic resource, enabling us to reveal the genetic mechanisms behind special properties, conduct evolutionary studies of the genus *Ehretia* and family Boraginaceae, and elucidate the molecular breeding of *E. macrophylla*.

## Methods

### Plant materials, library construction, and genome size estimation

**Fig. 1:**
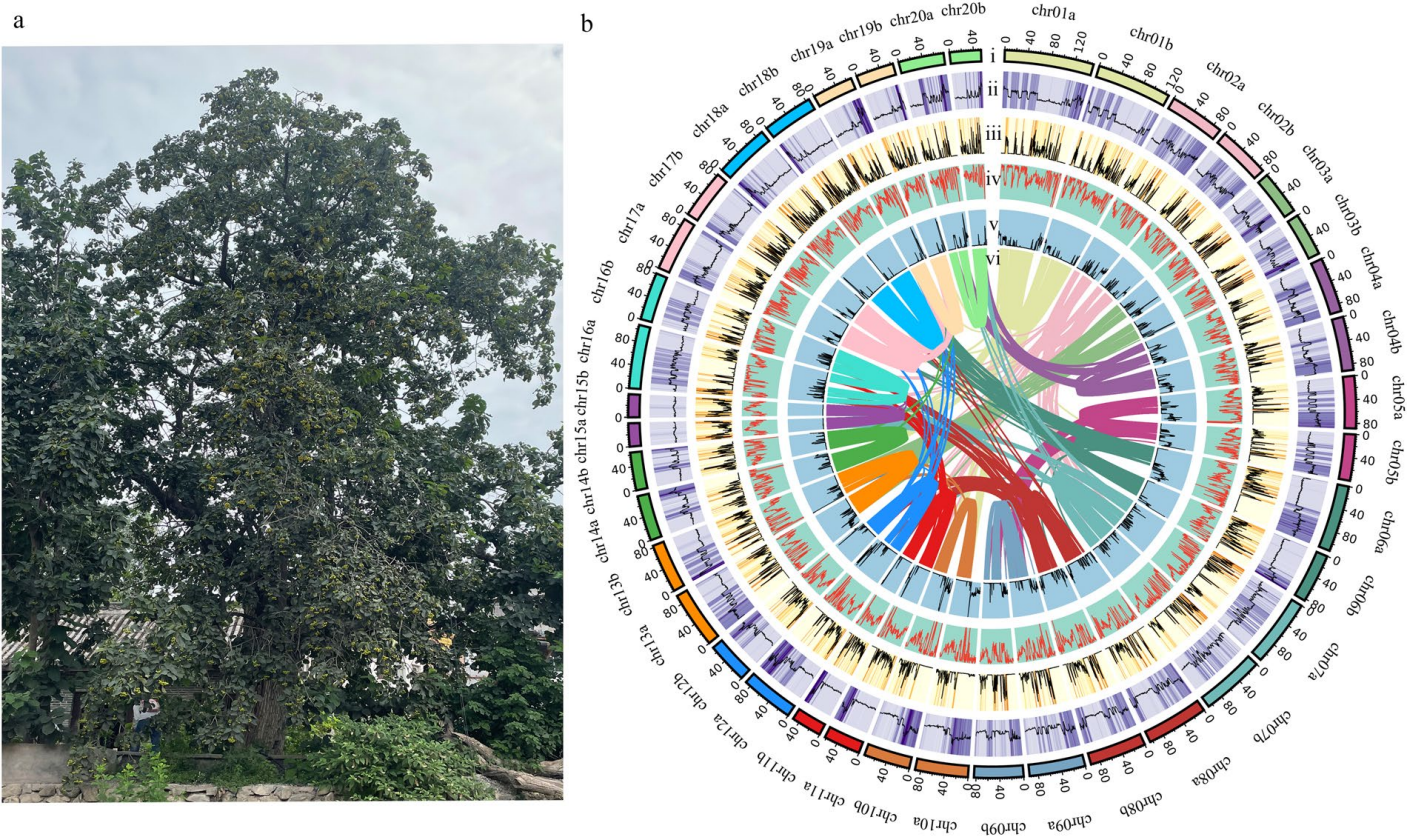
The morphological characteristics and features of the haplotype-resolved genome assembly of *E. macrophylla*. (**a**) The overall morphological characteristics of *E. macrophylla*; (**b**) An overview of haplotype-resolved genome assembly of *E. macrophylla*. (i) The distribution of pseudochromosomes, (ii) GC content, (iii) gene density, (iv) repeat density, (v) ncRNA, and (vi) analysis of collinearity.

Fresh leaf tissue for genome and RNA sequencing was sampled in 2022 from a mature *E. macrophylla* individual growing in Luoyang, Henan Province, China (34.663041 N, 112.434468 E) (Fig. 1a). Superior-quality genomic DNA was isolated using the Plant Genomic DNA Kit (Tiangen, China). The concentration and purity of the genomic DNA were assessed using a NanoDrop 8000 spectrophotometer (Thermo Fisher Scientific, USA). Total RNA was extracted from *E. macrophylla* samples utilizing TRIzol reagent. Subsequently, RNase-free DNase I was employed to treat the isolated RNA, followed by elution with RNase-free water. RNA integrity was measured using an Agilent 2100 Bioanalyzer (Agilent Technologies, Palo Alto, CA, USA).

The DNA that met the required qualifications was utilized to construct a genome library using the Pacific Biosciences SMRTbell Express Template Prep Kit. A 20-kb insert library was processed using a BluePippin system. The sequencing was carried out using the Pacific Bioscience Sequel II platform (Pacific Biosciences, Menlo Park, CA, USA). We obtained ~152.18 Gb of PacBio HiFi raw data (~84 × ) with an average length of 16.05 kb (Table 1). For Illumina sequencing, the sequencing was performed on the HiSeq X Ten platform (Illumina) with model of 150 PE. Finally, we obtained approximately 54.03 Gb of Illumina raw data (~30 × ). The Hi-C libraries were constructed, enriched, and sheared according to methods described previously^6,7^. The Hi-C sequencing was conducted using the Illumina HiSeq X Ten platform. A total of approximately 208.36 Gb (~114 × ) of raw Hi-C data were acquired. For RNA sequencing, a cDNA library was constructed using an RNA Library Prep Kit (NEB, UK). Approximately 9.66 Gb of raw data were obtained from the HiSeq X Ten platform (Illumina).

**Table 1:** The statistics of the genome sequencing data of *E. macrophylla*.

Pair-end libraries	Total data (Gb)	Average reads length (bp)	Sequence depth
Raw bases of WGS-PacBio HiFi	~152.18	16,050	~84×
Raw bases of WGS-Illumina	~54.03	149.5	~30×
Raw bases of Hi-C	~208.36	150	~114×
Raw bases of RNA-seq	~9.66	148.9	—
Total	~424.23	—	~228×

### Genome survey and assembly

**Fig. 2:**
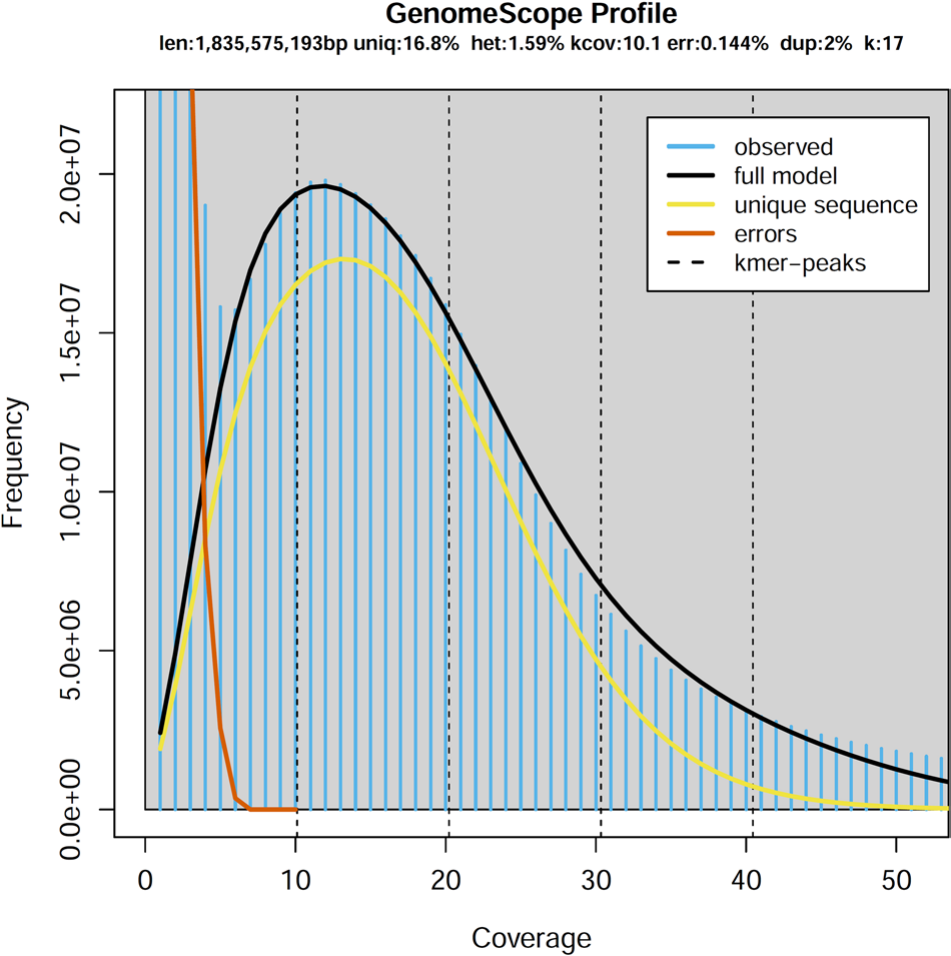
Overview of the 17-mer frequency distribution in the genome of *E. macrophylla*.

**Fig. 3:**
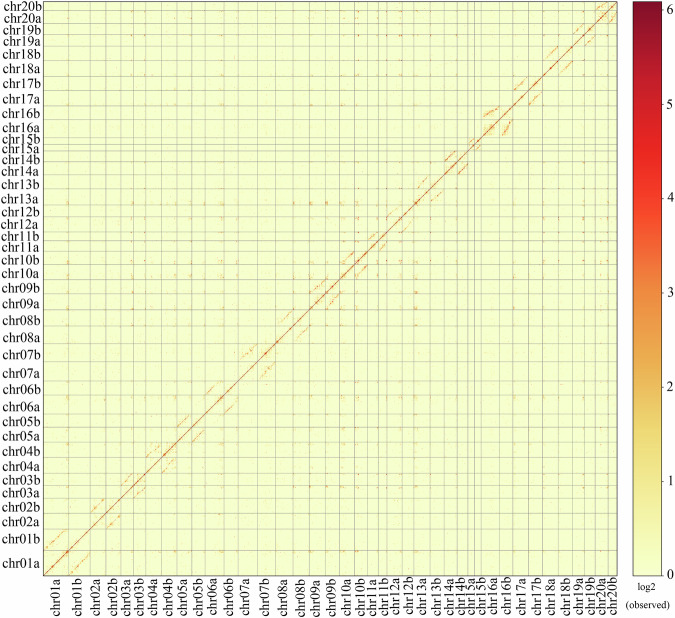
The Hi-C interaction heatmap of chromosome interactions in *E. macrophylla* chromosomes.

Before assembly, the adaptor sequences, low-quality regions, and sequences that were overly short were removed using the fastp v0.19.3^8^ software. Jellyfish v2.3.0^9^ was employed for determining the frequency distribution of the depth of clean data with 17 K-mers, and GenomeScope v2.0^10^ was utilized to estimate the genome size. The estimated haplotype genome size for *E. macrophylla* is approximately 1.84 Gb (Fig. 2). A combination of HiFi reads and Hi-C short reads was employed as input for the genome assembler Hifiasm v0.16.1^11^. The assembly process, conducted in Hi-C mode with default settings, resulted in the generation of two contigs representing haplotype a and haplotype b, respectively. For chromosome assembly, we first aligned the Hi-C reads to the assembly using Juicer v1.6 software^12^. Next, the draft genome assembly was scaffolded using 3D-DNA^13^ with Hi-C reads. Then, we manually adjusted the chromosome construction using the Juicebox tool^14^, which involved removing incorrect insertions and adjusting the orientation to correct visible errors to the best extent possible. For further optimization of the genome assembly, three rounds of corrections were performed on the assembly using Illumina reads with NextPolish v1.4.0^15^, and the redundant sequences were removed using Redundans v0.14a27^16^. In total, approximately 99.41% the assembled data was anchored onto 40 pseudochromosomes in the two haplotypes (Supplementary Table 1). Finally, we obtained a high-quality haplotype-resolved chromosomal-level genome of *E. macrophylla* (Fig. 1b, Fig. 3). The assembly (~3.40 Gb) comprised two haplotypes, namely haplotype a and haplotype b, with respective genome sizes of 1.82 Gb and 1.58 Gb (Table 2). Since the genome assembly was haplotype-resolved and lacked parental information for subgenome phasing, we designated the long one chromosome from each homologous pair as haplotype a and the other as haplotype b. The contig N50 and the scaffold N50 lengths for haplotype a were 28.11 Mb and 92.55 Mb, respectively, whereas for haplotype b, they were 21.57 Mb and 83.31 Mb, respectively. A total of 307 gaps were identified in the current genome assembly (Table 2). Utilizing PacBio HiFi reads, the LR_Gapcloser^17^ software was employed for gap filling, with two iterations executed. Furthermore, we assembled a chloroplast genome with a length of 156,639 bp and a mitochondrial genome with a length of 702,890 bp using GetOrganelle v1.7.5.0^18^.

**Table 2:** Summary of the *E. macrophylla* genome assembly data.

Statistic	Haplotype a		Haplotype b	
Total size (bp)	1,818,931,463		1,580,120,887	
Number of gaps	158		149	
GC content (%)	39.76		38.91	
Characterstic	Contig	Scaffold	Contig	Scaffold
Number	178	20	169	20
Maximum (bp)	82,539,829	151,004,203	66,459,732	126,475,925
Mean (bp)	10,218,629	90,946,573	9,349,741	79,006,044
Minimum (bp)	42,132	39,249,396	95,526	38,823,807
Median (bp)	4,342,499	92,548,202	4,276,896	82,072,805
N10 (bp)	57,407,540	113,720,351	47,364,060	106,967,081
N50 (bp)	28,110,220	93,350,056	21,565,141	83,310,942
N90 (bp)	4,361,587	74,743,574	4,635,546	64,090,437
L10	3	2	3	2
L50	23	9	24	9
L90	88	17	81	17

### Genomic repeat annotation

To annotate the repeat sequences in the *E. macrophylla* genome, a transposable element (TE) library was first constructed by running the extensive *de novo* TE Annotator (EDTA) pipeline to identify TEs from scratch. The parameters used were–Sensitive 1–ANNO 1^19^. Then, we used RepeatMasker v4.1.3 (http://www.repeatmasker.org/RepeatMasker/) to mask the repeat library acquired from the Repbase database (https://www.girinst.org/repbase/). For *E. macrophylla* haplotype a, a total of 2,751,291 repetitive sequences, constituting approximately 79.18% of the genome, were identified with a cumulative length of 1.44 Gb. Among them, long terminal repeats (LTRs) were the main repeats, totaling 851,702, with a size of 790.85 Mb, accounting for 43.48% of the assembled genome. This was followed by DNA transposable elements (TIRs) at 29.36%. The sizes of the copia- and gypsy-like LTRs were 109.80 Mb and 351.66 Mb, respectively, which accounted for 6.04% and 19.33% of haplotype a (Table 3). In term of *E. macrophylla* haplotype b, a total of 2,258,809 repetitive sequences (76.29% of the genome) were identified with a length of 1.21 Gb. Of these, the primary repetitive elements were also LTRs, which amounted to 788,470 and occupied a total size of 713.16 Mb, representing 45.13% of the genome that was assembled. This was followed by TIRs, accounting for 24.32%. The copia- and gypsy-like LTRs had sizes of 109.44 Mb and 314.71 Mb, respectively, making up 6.93% and 19.92% of haplotype b (Table 3).

**Table 3:** The repetitive sequences identified in the genome of *E. macrophylla*.

Order	Superfamily	Number	Length (bp)	Percent (%)
**Haplotype a**				
LTR		851,702	790,852,679	43.48
	Copia	135,351	109,800,558	6.04
	Gypsy	285,814	351,659,875	19.33
	Retrovirus	314	86,793	—
	unknown	430,223	329,305,453	18.10
LINE		5,195	3,078,252	0.17
TIR		1,199,599	534,061,496	29.36
	EnSpm_CACTA	117,091	50,176,752	2.76
	MuDR_Mutator	291,572	91,979,798	5.06
	PIF_Harbinger	19,645	6,162,896	0.34
	Tc1_Mariner	7,691	1,968,931	0.11
	hAT	763,600	383,773,119	21.10
Helitron		63,169	16,361,313	0.90
	Helitron	63,169	16,361,313	0.90
Other TEs		631,626	95,801,905	5
Total TEs		2,751,291	1,440,155,645	79.18
**Haplotype b**				
LTR		788,470	713,166,126	45.13
	Copia	136,732	109,438,952	6.93
	Gypsy	259,567	314,706,790	19.92
	Retrovirus	335	99,704	0.01
	unknown	391,836	288,920,680	18.28
LINE		5,175	2,974,698	0.19
TIR		841,681	384,318,183	24.32
	EnSpm_CACTA	108,323	46,824,686	2.96
	MuDR_Mutator	249,750	79,863,483	5.05
	PIF_Harbinger	19,800	6,193,662	0.39
	Tc1_Mariner	7,631	1,978,503	0.13
	hAT	456,177	249,457,849	15.79
Helitron		63,141	16,196,919	1.03
	Helitron	63,141	16,196,919	1.03
Other TEs		560,342	88,821,425	6
Total TEs		2,258,809	1,205,477,351	76.29

### Gene identification and functional annotations

To annotate the high-quality protein-coding genes, a comprehensive approach encompassing homology-based, *de novo*, and transcriptome-based predictions was employed. A total of 31,9767 non-redundant protein sequences from closely related species (*Echium plantagineum*^20^, *Solanum lycopersicum*^21^, *Coffea canephora*^22^, *Eucommia ulmoides*^23^, *Tectona grandis*^24^, *Daucus carota*^25^, *Nyssa sinensis*^26^, *Rhododendron simsii*^27^, *Lonicera japonica*^28^, *Lactuca saligna*^29^, *Vitis vinifera*^30^, and *Arabidopsis thaliana*^31^) were gathered as evidence for protein homology using Exonerate V2.4.0^32^. The RNA-seq data were aligned to the genome sequences using Hisat2 v2.2.0^19^ with default parameters, followed by assembly of the aligned reads using StringTie 2 v2.1.2^33^. Subsequently, all splicing variations were identified and classified through alignment of full-length transcripts utilizing the PASA v2.3.3^34^ pipeline. All complete gene structures predicted using PASA v2.3.3 pipeline were utilized to generate a training model with AUGSTUS v3.3.3^35^, employing default parameters.

In addition, the putative protein-coding gene structure was predicted utilizing MAKER2^36^. The *ab initio* predictions of gene structure were conducted using AUGSTUS v3.3. We aligned the transcript evidence with the genome using BLAST+^37^ and finally optimized it with Exonerate v2.4.0^32^. In order to increase the accuracy of the annotation, we integrated and updated the gene prediction results using EVidenceModeler51 (EVM)^38^ and PASA. In total, we annotated 29,805 protein-coding genes in *E. macrophylla* haplotype a with an average length of 4,956.40 bp. Among them, there are a total of 36,131 coding DNA sequence (CDS), 200,786 exons, and 164,655 introns. The average lengths were 1,243.30 bp for CDS, 281 bp for exons and 803 bp for introns (Table 4). Additionally, we identified 29,081 protein-coding genes in haplotype b a with an average length of 5,199.10 bp. A total of 34,686 CDS, 191,925 exons, and 157,239 introns were detected, with the average lengths of 1,248.6 bp, 279.1 bp and 854.6 bp respectively (Table 4).

**Table 4:** Statistical analysis of gene annotations.

Category	Feature	Haplotype a		Haplotype b	
		Number	mean	Number	mean
**All the genes**	Gene	449,771	467.7	278,024	694.4
	Transcript	456,097	261.1	283,629	336.5
	CDS	36,131	1,243.3	34,686	1,248.6
	Exon	620,805	191.8	440,921	216.4
	Intron	164,708	802.7	157,292	854.3
	Exons/transcript	456,097	1.4	283,629	1.6
**Coding genes**	Gene	29,805	4,956.4	29,081	5,199.1
	Transcript	36,131	1,561.6	34,686	1,544.3
	CDS	36,131	1,243.3	34,686	1,248.6
	Exon	200,786	281	191,925	279.1
	Intron	164,655	803	157,239	854.6
	Exons/transcript	36,131	5.6	34,686	5.5

Functional annotation of protein-coding genes was carried out using three strategies. First, we mapped gene sequences against the eggNOG 5.0^39^ database using eggNOG-mapper v2.16^40^, and annotated 97.94% of the genes. Of these 48.80% and 47.94% were annotated with Gene Ontology (GO, http://geneontology.org/) and Kyoto Encyclopedia of Genes and Genomes (KEGG, https://www.genome.jp/kegg), respectively. Second, 98.40% of genes were annotated using DIAMOND v2.0.12^41^ against four protein databases: Swiss_Prot^42^ (78.96%), TrEMBL^42^ (98.39%), NR^43^ (98.23%), and *Arabidopsis thaliana* genes (91.53%). Finally, InterProScan v5.5.2-86.0^44^ was used to annotate 98.74% of the gene against 14 databases (Table 5).

**Table 5:** Statistics of protein-coding gene functional annotation for *E.macrophylla*.

Method	Database	Number	Percent (%)
eggNOG-mapper		57,674	97.94
	GO	28,739	48.80
	KEGG_KO	28,231	47.94
	EC	13,170	22.37
	KEGG_Pathway	18,553	31.51
	eggNOG	54,355	92.31
	COG	57,674	97.94
DIAMOND		57,971	98.40
	Swiss_Prot	46,499	78.96
	TrEMBL	57,936	98.39
	NR	57,846	98.23
	*A.thaliana*	53,901	91.53
InterProScan		58,146	98.74
	Pfam	49,328	83.77
	Coils	8,957	15.21
	SUPERFAMILY	38,859	65.99
	PIRSF	4,037	6.86
	PANTHER	56,540	96.02
	Gene3D	41,006	69.64
	Phobius	21,112	35.85
	PRINTS	9,861	16.75
	TIGRFAM	5,870	9.97
	CDD	19,867	33.74
	Interpro	52,076	88.44
	MobiDBLite	21,643	36.75
	TMHMM	15,261	25.92
	SMART	17,398	29.55
Total		58,650	99.60

For the annotation of non-coding RNA genes, we detected a total of 415,016 rRNA genes, 2,522 tRNA genes, and 2,428 other ncRNA genes in haplotype a using tRNAScan-SE^45^, Barrnap (https://github.com/tseemann/barrnap), and Rfam^46^, respectively. In term of haplotype b, a total of 244,274 rRNA genes, 2,409 tRNA genes, and 2,260 other ncRNA genes were detected (Table 6).

**Table 6:** Statistics for non-coding RNA genes in the genome of *E. macrophylla*.

RNA genes	Haplotype a	Haplotype b	Total
rRNA	415,016	244,274	659,290
tRNA	2,522	2,409	4,931
Other ncRNA	2,428	2,260	4,688

### Genome comparison between haplotype assemblies

**Fig. 4:**
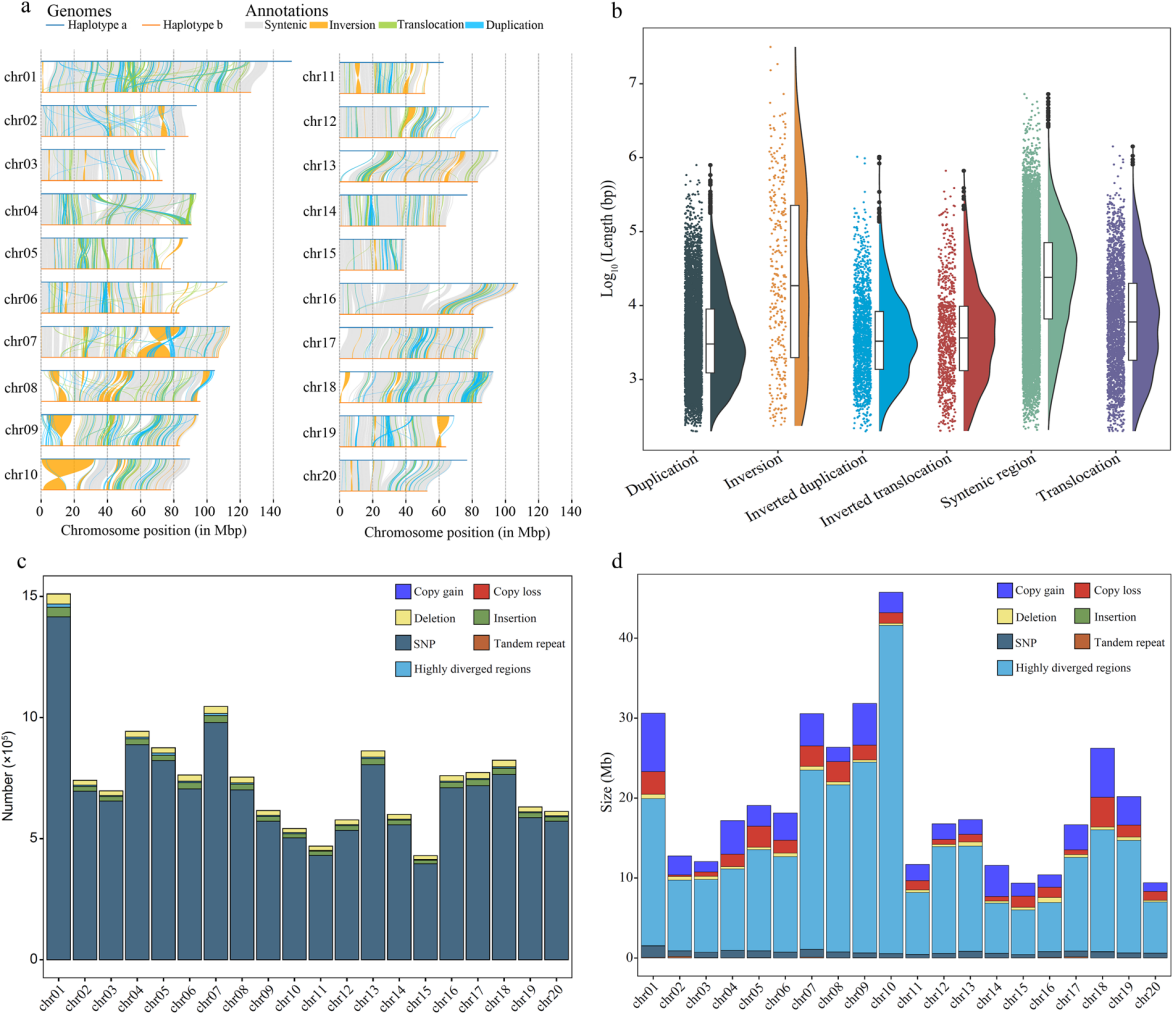
Structural variation and statistics between two haplotype genome assemblies of *E. macrophylla*. (**a**) Structural variation between haplotype genomes (haplotype a and haplotype b). (**b**) Size distributions of different types of structural variation between haplotype a and haplotype b. (**c**) Number of sequence differences in the syntenic region for each pair of chromosomes. (**d**) Size of sequence differences in the syntenic region for each pair of chromosomes.

**Fig. 5:**
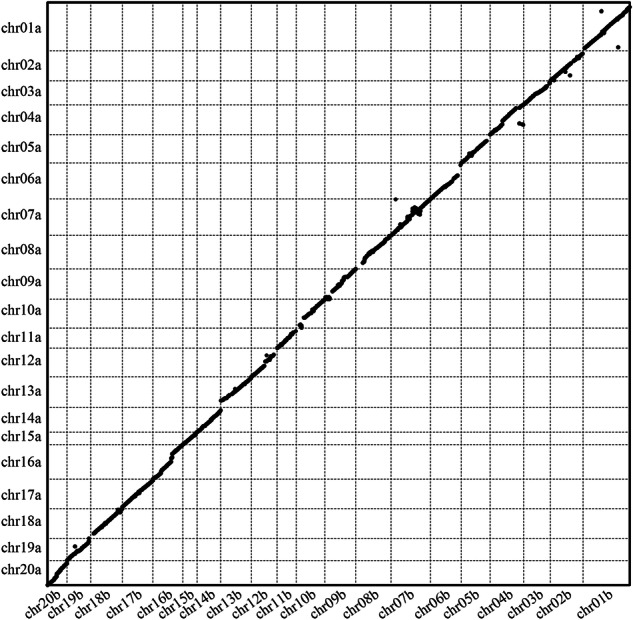
Dot-plot of synteny blocks between the two haplotypes within *E. macrophylla*.

The haplotype alignments were conducted utilizing minimap2^47^, while the identification of syntenic regions and structural variations was performed using SyRI v1.6^48^. The structural rearrangements identified between haplotype genomes were visualized using Plotsr v0.5.4^49^ (Fig. 4). Chr 01, 02, and 04 to 10 exhibit more structural variation (Fig. 4a). A total of 13,045 syntenic regions (~953 Mbp) were detected, indicating extreme similarity between the two haplotypes (Fig. 4b). Numerous variations were also detected, including minor insertions/deletions and SNPs (Fig. 4c,d); two relatively large inversions were found on chr07 and chr10, respectively (Fig. 4a). We compared the dot plot of syntenic blocks using Minimap2 and found that the two haplotypes were very similar, with essentially the same chromosome order (Fig. 5).

## Data Records

The sequencing data for this study have been uploaded to the NCBI database with the BioProject number PRJNA945189. The genomic PacBio sequencing data can be found in the NCBI Sequence Read Archive (SRA) database with accession numbers SRR23907027^50^, SRR23907028^51^, SRR23907029^52^, and SRR23907030^53^. For Hi-C sequencing data, specifically referring to accession numbers SRR23907031^54^ and SRR23907036^55^ in the SRA database. The genomic Illumina sequencing data are available under accession numbers SRR23907047^56^ and SRR23907058^57^. The final genome assembly was deposited in the GenBank with accession number: GCA_037974685.1^58^ and GCA_037974665.1^59^. In addition, the final chromosome assembly and annotation data were deposited in the Genome Warehouse (GWH) of the National Genomics Data Center (NGDC) with the accession number GWHEQHN00000000^60^ and under the BioProject number PRJCA021125.

## Technical Validation

**Fig. 6:**
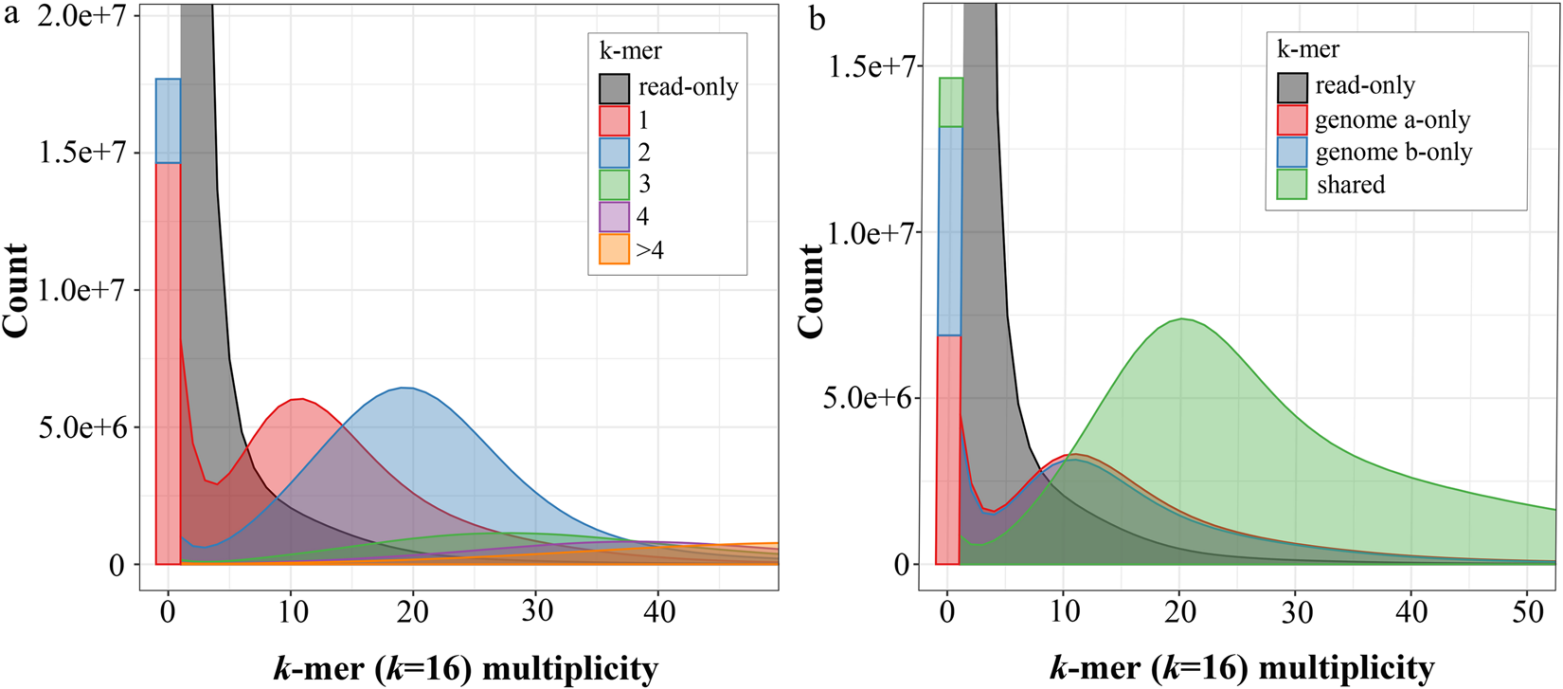
Evaluation of genome quality utilizing the Merqury spectrum plot. (**a**) Spectrum plot demonstrating copy number variations in haplotype assemblies of *E. macrophylla*. (**b**) Spectrum plot for assessing the completeness of K-mer assembly.

**Fig. 7:**
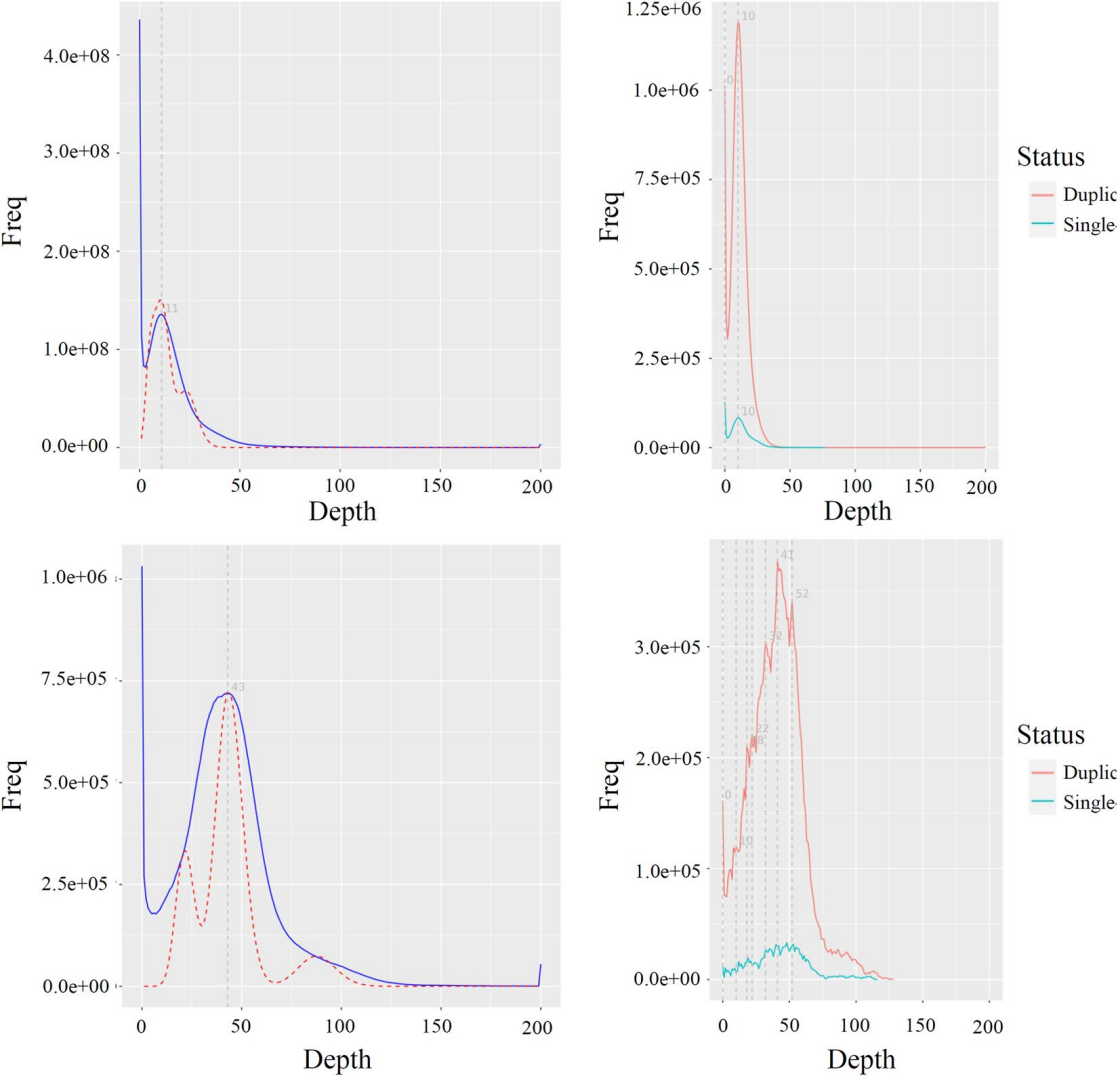
The coverage depth of the genome (left) and the distribution of BUSCO core regions (right) assessed using the next-generation data (upper) and HiFi data (lower).

To evaluate the completeness and accuracy of the genome, we employed BWA^61^, minimap2^47^, and HISAT2^19^ to align Illumina reads, HiFi reads, and RNA-Seq reads to our reference genome respectively. In addition, BUSCO v5.2.2^62^ was used to evaluate the genome completeness using the embryophyta_odb10 and eukaryota_odb10 databases. The genomic completeness of these two haplotypes was found to be satisfactory, with proportions of complete BUSCOs (including both single-copy and multi-copy) at 98.1% and 97.1% for the expected genes from embryophyta, respectively (Table 7). The *E. macrophylla* genome size was evaluated using k-mer analysis (Fig. 2). After filtering out non-primary alignments, we proceed to calculate the mapping ratio and coverage percentage. We found that the genome coverage from sequencing data is relatively high (Table 8). We conducted additional quality control analysis on the genome assembly using Merqury^63^ (at K = 16) based on PacBio HiFi reads (Fig. 6, Table 9). The consensus quality values (QVs) of the separate haplotypes a and b, as well as their shared genome, are recorded as 34.98, 34.74, and 34.87 correspondingly. The k-mer completeness scores of the distinct haplotypes a and b, along with their shared genome, amount to approximately 82.08%, 81.07%, and 94.46% accordingly. The further BUSCO analysis showed that the single-copy and multi-copy genes have approximately the same depth, indicating that the assembly had no redundancy (Fig. 7).

**Table 7:** Statistical analysis of BUSCO for both haplotypes and proteins.

Description	BUSCO groups		
	Haplotype a	Haplotype b	Proteins
Complete BUSCOs	1,583 (98.1%)	1,574 (97.5%)	1,556 (96.4%)
Complete and single-copy BUSCOs	1,486 (92.1%)	1,479 (91.6%)	114 (7.1%)
Complete and duplicated BUSCOs	97 (6.0%)	95(5.9%)	1,442 (89.3%)
Fragmented BUSCOs	24 (1.5%)	21 (1.3%)	14 (0.9%)
Missing BUSCOs	7 (0.4%)	19 (1.2%)	44 (2.7%)
Total BUSCO groups searched	1,614	1,614	1,614

**Table 8:** Statistics of map rate and coverage of three types of sequencing reads.

Data set	Illumina seq	HiFi seq	RNA-seq
Reads mapped (%)	99.90	99.90	93.70
Properly paired (%)	91.90	—	89.20
Bases mapped (%)	99.90	99.90	93.80
≥1 × (%)	87.20	97.00	4.50
≥5 × (%)	76.40	94.50	2.40
≥10 × (%)	59.50	91.80	1.80
≥20 × (%)	25.30	84.60	1.30

**Table 9:** Statistical analysis of Merqury for evaluating the quality of haplotypes.

Assembly	QV (quality value)	Error rate	Completeness (%)
Haplotype a	34.98	3.18e-04	82.08
Haplotype b	34.74	3.36e-04	81.07
Haplotype both a and b	34.87	3.26e-04	94.46

**Fig. 8:**
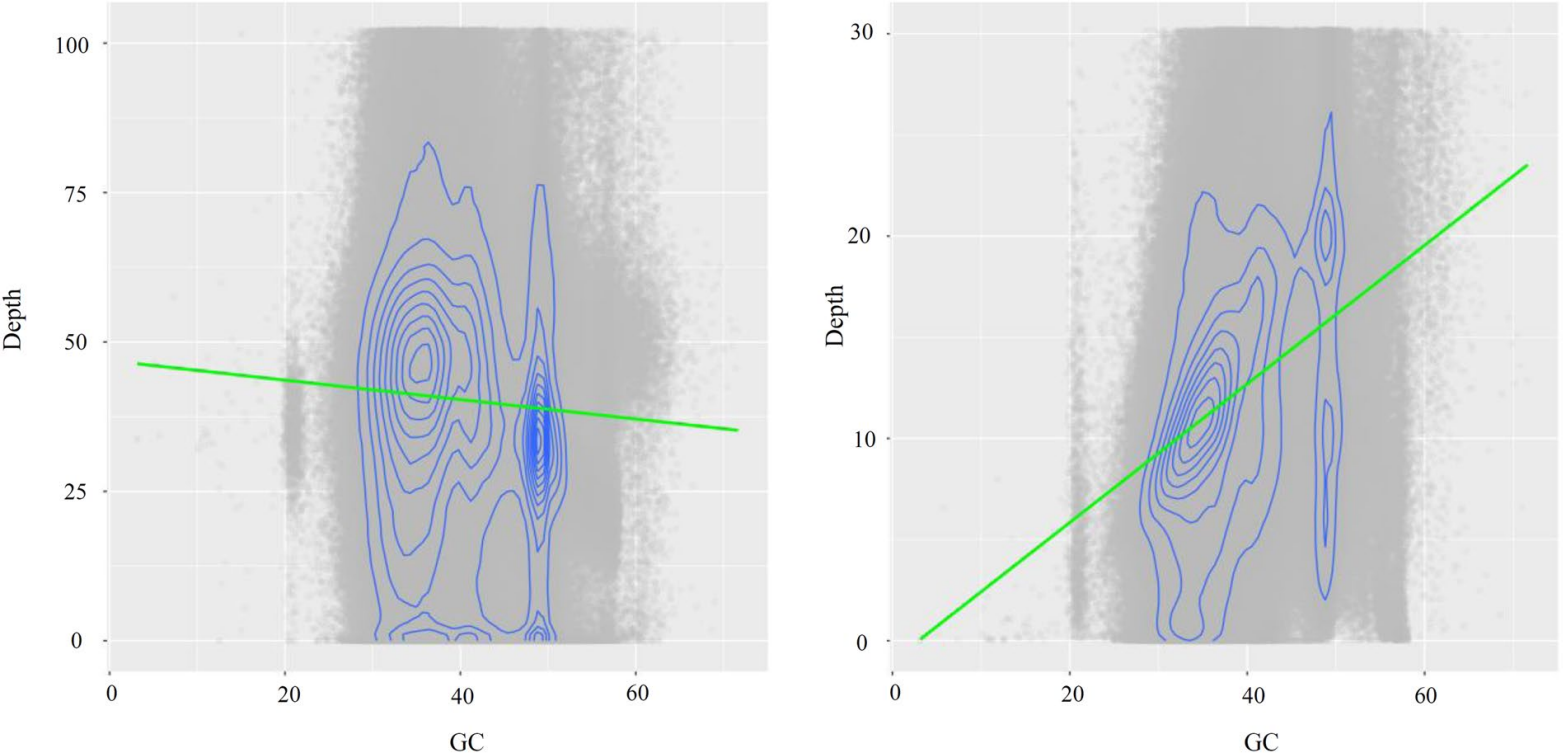
The coverage depth for HiFi data (right) and next-generation data (left).

To evaluate the single-base error rate and heterozygosity, next-generation reads were mapped to the genome using BWA, and the variant loci were detected using bcftool v 1.11^64^. Heterozygous sites were utilized for the computation of heterozygosity rates, whereas homozygous sites were employed for the determination of error rates. We found that the heterozygosity rate was approximately 0.19%, and the error rate was approximately 0.012%. By evaluating the coverage depth and GC content distribution analysis of the second and third generation data, we found that the second-generation data had a significant guanine-cytosine (GC) bias (Fig. 8). Juicer^12^ was used to map the Hi-C data to the final genome assembly. It was found that the chromosome clustering was normal, with no obvious chromosome assembly errors, but there were abnormal signals in some regions (Fig. 3). The chromatin interaction data from the Hi-C map revealed low-level interactions occurred between pseudochromosomes, confirming the high quality and reliability of our chromosome-level anchoring (Supplementary Table 1).

The chromosomal locations of specific characteristic sequences, such as telomeres, rDNA, and tandem repeats, were determined through the mapping of repetitive sequences onto the genome. The majority of chromosome telomere sequences were completely assembled; however, a few exhibited partial or missing regions. We detected a high tandem repeat on chromosomes (Supplementary txt 1). This sequence contains 5 S rDNA, and its distribution is essentially consistent, suggesting that this sequence represents 5 S rDNA and its adjacent regions. In addition, the 18-5.8-28 S rDNA and 5 S rDNA arrays are very abundant and widely distributed (Supplementary Fig. 1).

BUSCO v5.2.2^62^ was employed to assess the annotated and integrated proteins utilizing the embryophyta_odb10 and eukaryota_odb10 databases. The proportion of complete core gene coverage was 96.4% (Table 7), which included 7.1% single-copy genes and 89.3% duplicated genes. Only 0.9% fragmented and 2.7% missing genes were detected, indicating that the genome annotation is of superior quality.

## Supplementary information

Supplementary information

## Data Availability

All software and pipelines were executed in accordance with the manual and protocols of the published bioinformatics tools, adhering to the specified versions and meticulously documenting the code/parameters used, as elaborated in the Methods section.
