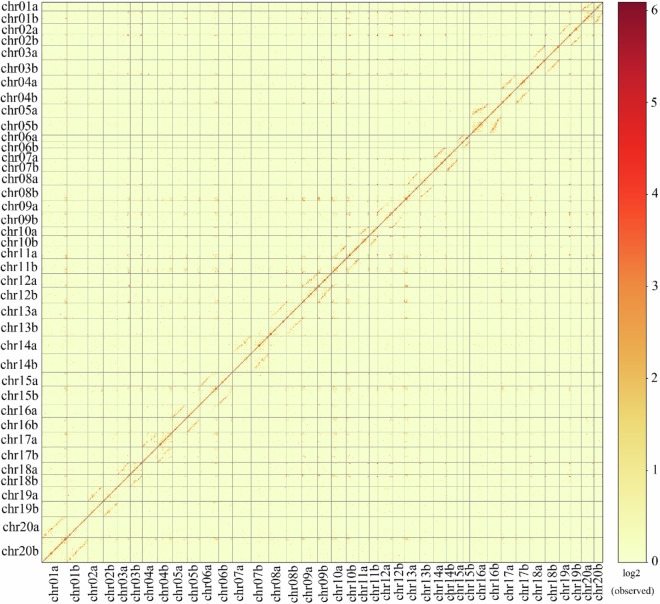# Author Correction: Haplotype-resolved chromosome-level genome assembly of *Ehretia macrophylla*

**DOI:** 10.1038/s41597-024-03791-2

**Published:** 2024-10-01

**Authors:** Shiping Cheng, Qikun Zhang, Xining Geng, Lihua Xie, Minghui Chen, Siqian Jiao, Shuaizheng Qi, Pengqiang Yao, Mailin Lu, Mengren Zhang, Wenshan Zhai, Quanzheng Yun, Shangguo Feng

**Affiliations:** 1 Henan Province Key Laboratory of Germplasm Innovation and Utilization of Eco-economic Woody Plant, Pingdingshan University, Pingdingshan, 467000 China; 2 Kaitai-bio Company, Hangzhou, 310000 China; 3 Henan Forestry Vocational College, Luoyang, 471000 China; 4 Henan Senzhuang Cukang Agriculture and Forestry Technology Co., Ltd, Luoyang, 471000 China; 5 Kaitai Mingjing Genetech Corporation, Beijing, China; 6 College of Life and Environmental Science, Hangzhou Normal University, Hangzhou, 310036 China; 7 Zhejiang Provincial Key Laboratory for Genetic Improvement and Quality Control of Medicinal Plants, Hangzhou Normal University, Hangzhou, 310036 China

**Keywords:** Genome, Evolutionary genetics

Correction to: *Scientific Data* 10.1038/s41597-024-03431-9, published online 05 June 2024

In Fig. 1b of this article, the number of chromosome20 of haplotype b were incorrectly given as chr21b but should have been chr20b. The vertical coordinates in Fig. 3 of the article are erroneously labeled as chr01a, chr01b… to chr20a, chr20b from top to bottom. The correct order should be from top to bottom: chr20b, followed by chr20a… to chr01b, and finally chr01a. The original and corrected Fig. 1 and Fig. 3 are shown below. The original article has been updated.

The corrected version of Fig. 1 is:
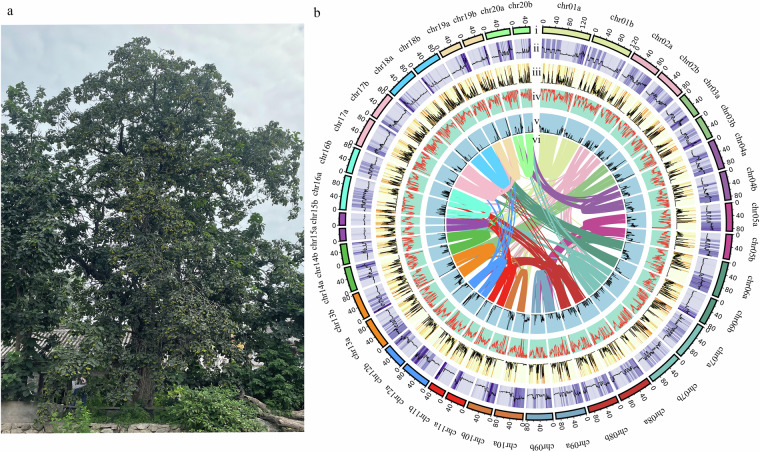


The original version of Fig. 1 is:
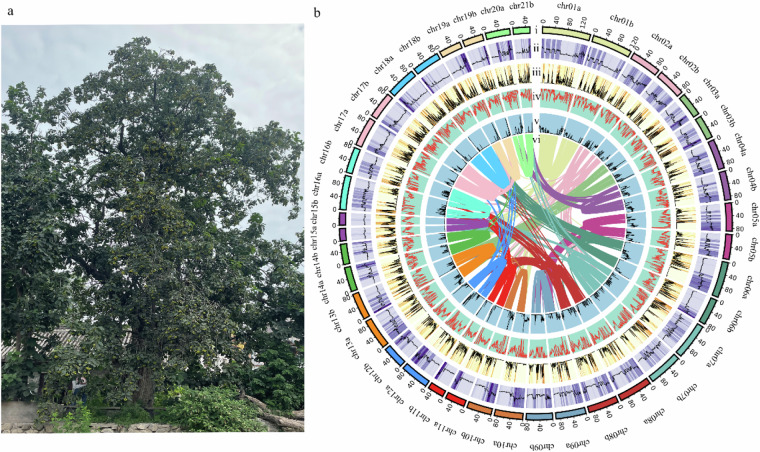


The corrected version of Fig. 3 is:
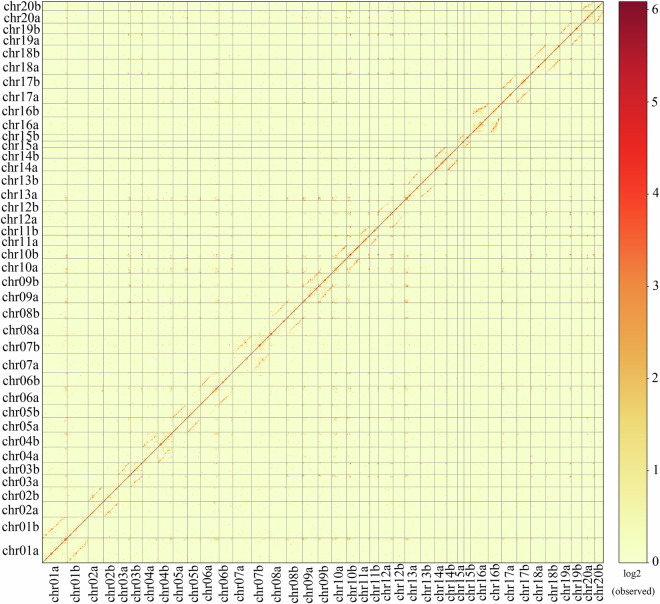


The original version of Fig. 3 is: