# Supplementary material for: Haplotype-resolved chromosome-level genome assembly of Ehretia macrophylla
Source: Sci Data. 2024 Jun 5;11:589. doi: 10.1038/s41597-024-03431-9 (PMC11153487; doi:10.1038/s41597-024-03431-9)
Supplement: Supplementary file 1 — Supplementary information [file 41597_2024_3431_MOESM1_ESM.docx]

**Supplementary information for**

**‘Haplotype-resolved chromosome-level genome assembly of *Ehretia macrophylla*’**

| **Content** | **Page** |
| --- | --- |
| Supplementary Table 1 | 1 |
| Supplementary txt 1 | 2 |
| Supplementary Fig. 1 | 3 |

**Supplementary Table**

**Supplementary Table 1.** Statistics on the chromosomal-level assembly of *E. macrophylla*.

| **Chr ID** | **Length (bp)** | **Percent (%)** |
| --- | --- | --- |
| chr01a | 151,004,203 | 8.30 |
| chr01b | 126,475,925 | 8.00 |
| chr02a | 93,742,786 | 5.15 |
| chr02b | 88,729,660 | 5.62 |
| chr03a | 74,743,574 | 4.11 |
| chr03b | 73,154,379 | 4.63 |
| chr04a | 93,350,056 | 5.13 |
| chr04b | 90,714,787 | 5.74 |
| chr05a | 88,494,918 | 4.87 |
| chr05b | 78,181,282 | 4.95 |
| chr06a | 112,240,985 | 6.17 |
| chr06b | 83,316,270 | 5.27 |
| chr07a | 113,720,351 | 6.25 |
| chr07b | 106,967,081 | 6.77 |
| chr08a | 104,709,135 | 5.76 |
| chr08b | 96,038,216 | 6.08 |
| chr09a | 94,740,387 | 5.21 |
| chr09b | 83,567,920 | 5.29 |
| chr10a | 89,603,608 | 4.93 |
| chr10b | 78,258,017 | 4.95 |
| chr11a | 62,609,922 | 3.44 |
| chr11b | 51,630,492 | 3.27 |
| chr12a | 89,877,233 | 4.94 |
| chr12b | 69,821,109 | 4.42 |
| chr13a | 95,602,259 | 5.26 |
| chr13b | 83,310,942 | 5.27 |
| chr14a | 76,914,456 | 4.23 |
| chr14b | 64,090,437 | 4.06 |
| chr15a | 39,249,396 | 2.16 |
| chr15b | 38,823,807 | 2.46 |
| chr16a | 107,478,667 | 5.91 |
| chr16b | 80,915,008 | 5.12 |
| chr17a | 92,480,322 | 5.08 |
| chr17b | 83,230,602 | 5.27 |
| chr18a | 92,616,081 | 5.09 |
| chr18b | 85,864,368 | 5.43 |
| chr19a | 68,912,987 | 3.79 |
| chr19b | 64,118,344 | 4.06 |
| chr20a | 76,840,137 | 4.22 |
| chr20b | 52,912,241 | 3.35 |
| Total | 3,399,052,350 (99.41%) | — |
| Mt | 702,890 (0.02%) | — |
| Pt | 156,639 (0.00%) | — |
| Tg | 13,669,239 (0.40%) | — |

Note: Mt: Mitochondria genome; Pt: Plastid genome; Tg: Scattered sequence

**Supplementary txt**

>**Supplementary txt 1** The characteristic unit sequence of the highly tandem, repetitive sequences distributed on chromosomes.

GATCAAAAGTTATGGGCACCGGAGCGACGGAGACCTGGTCACAAGCCCTACGCCCTCGGTTTGCGGGCCCAGCCTCTACGTGAAGCGAAGTTGTCTTGCCCAAGGTGCCGTGGGATTCGATAATAGAAATGTGGTGGATAAAGTATCTTGGCAACCCTAACCGGAGCAAAAAAAATTGAAAAGGGGGGTGCAACACGAGGACTTCCCAGGGGGTCACCCATCCTAGTACTACTCTCGCCCAAGCACGCTTAACTTCGGAGTTCTGATGGGATCCGGTGCATTAGTGCTGGTATGATCGCACCCGTCATGTTGAGGGCACAATTCCTTTATGTGTTAACGGTTCACAGTCCCAGCCTAGAGAGATGGCCCATTCAAAAGGCCCAATGGGCTTAACGACAACCCATCCGCTTCAACAACGCAGCATCCTCGCAAGACGTGATATCGACGCATTGTAGGGTCCTCTAATGTGCCGCGAGTAAGGTTGTCGTCCTTTTAACAGCTCACGACGTACGATCCGGGACGGAAAAACAGAGCAAAAGAAGAAAAGTGGCCCGAAACTGATGAAAACGTTCGATTTTGGGCTCCAAAAAGGGCACAGCGAGCAAGCTGGCTGCCCCCGTCGTAGAGCTCTCGGAGAGCTTTCCAACGGTATGCTGTGCGCCGCGTAACTCCTTACG

**Supplementary Figure**


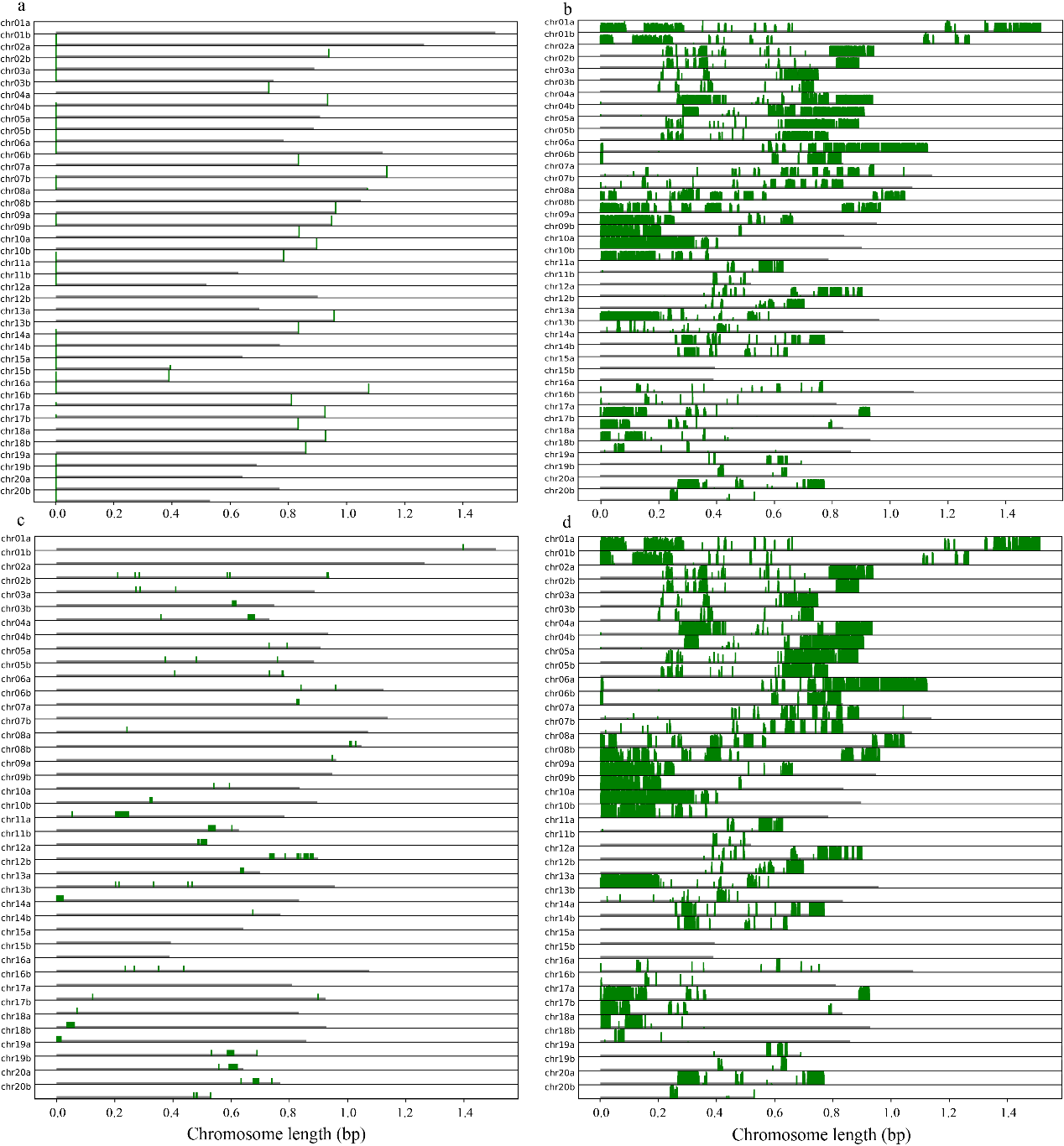


**Supplementary Fig. 1.** Bar plot showing the density and distribution of several repeated elements on the chromosome sequences of *E. macrophylla*. (a) Telomeres. (b) Tandem repeats. (c) 18-5.8-28S rDNA. (d) 5S rDNA.
